# Impact of Seminal Chemical Elements on the Oxidative Balance in Bovine Seminal Plasma and Spermatozoa

**DOI:** 10.1155/2013/125096

**Published:** 2013-09-09

**Authors:** Eva Tvrdá, Norbert Lukáč, Monika Schneidgenová, Jana Lukáčová, Csaba Szabó, Zofia Goc, Agnieszka Greń, Peter Massányi

**Affiliations:** ^1^Department of Animal Physiology, Faculty of Biotechnology and Food Sciences, Slovak University of Agriculture, Tr. A. Hlinku 2, 949 76 Nitra, Slovakia; ^2^Department of Animal Physiology and Health, Szent István University, Páter Karoly utca 1, Gödöllő 2103, Hungary; ^3^Institute of Biology, Faculty of Geography and Biology, Pedagogical University of Krakow, ul. Podbrzezie 3, 31 045 Kraków, Poland

## Abstract

Mutual relationships between selected chemical elements (Na, K, Fe, Cu, Mg, and Zn), basic motility characteristics (motility and progressive motility), and markers of the oxidative balance (superoxide dismutase, catalase, glutathione, albumin, and malondialdehyde) were investigated in bovine seminal plasma and spermatozoa. Computer assisted sperm analysis was used to assess the motility parameters; mineral concentrations were determined by the voltammetric method and flame absorption spectrophotometry; antioxidants and malondialdehyde were evaluated by UV/VIS spectrophotometry. Concentrations of chemical elements in both seminal fractions were in the following descending order: Na > K > Zn > Mg > Fe > Cu. Higher amounts of all minerals and nonenzymatic antioxidants were detected in the seminal plasma (*P* < 0.01; *P* < 0.001), while higher MDA concentration and activity of enzymatic antioxidants were recorded in the cell lysates (*P* < 0.01; *P* < 0.001). Na, Fe, Cu, Mg, and Zn were positively correlated with the motility and antioxidant parameters (*P* < 0.05; *P* < 0.01; *P* < 0.001). Inversely, K exhibited the positive associations with malondialdehyde (*P* < 0.05). This study demonstrates that most chemical elements are integral components of bovine semen and are needed for the protection against oxidative stress development.

## 1. Introduction

Various anthropogenic activities and natural environmental factors as well as other sources are of crucial importance for the reproductive potential of semen, both in animals and humans [1]. Chemical elements represent a vital ecophysiological component for the preservation and fertilization capacity of spermatozoa. Some of them are essential for proper sperm cell functions (e.g., sodium, Na; potassium, K; calcium, Ca; magnesium, Mg); others are required in relatively narrow limits (e.g., zinc, Zn; copper, Cu; manganese, Mn; cobalt, Co; selenium, Se; iron, Fe) [2, 3].

Mammalian seminal plasma and spermatozoa are known to contain a broad variety of macro- and microelements [1]. The influence of major biologically active inorganic components on spermatozoa viability parameters has been studied in animals as well as in humans [2–7]. Positive effects on the sperm cell motility, morphology, and concentration were reported particularly for Zn, Mg, Se, and Ca [4, 5, 7]. Fe, Cu, and their compounds are essential metal cofactors for a variety of bioactive molecules; however, disturbances in their regulative absorption mechanism with subsequent aberrant concentrations may have a negative impact on the sperm viability and morphology [2, 3, 8].

The role of chemical elements in natural antioxidant structures has recently attracted much scientific interest. Some minerals are required for cellular defense systems against free radicals (FR) [1]. It has been demonstrated that disturbances in their concentrations may lead to a reduction of antioxidant activities and subsequently increase the risk of oxidative stress (OS) development [1, 6]. OS is a serious condition as FR, and their metabolites attack vital biomolecules including DNA, lipids, and proteins, altering enzymatic systems and cell signaling pathways and causing irreparable damage, apoptosis or necrosis [9]. The unique cellular architecture of spermatozoa, large quantities of polyunsaturated fatty acids, and low concentrations of FR scavengers render them to be particularly susceptible to OS. Meanwhile, the seminal plasma is an important protectant of spermatozoa against possible FR formation and distribution, as it contains an array of enzymatic as well as nonenzymatic antioxidants [10, 11].

A variety of data concerning the relationships between chemical elements, and antioxidant profile in semen have been currently published; however, the information is partial and related to whole semen or seminal plasma only. To our knowledge, no study is available on the relationships between seminal quality, chemical elements and markers of antioxidant status in both fragments of the ejaculate—the seminal plasma and spermatozoa fraction. 

This study was therefore carried out to (1) evaluate selected chemical elements (Na, K, Cu, Fe, Zn, and Mg) and markers of oxidative balance in bovine seminal plasma and spermatozoa and to (2) assess their mutual relationships as well as effects on the sperm motility characteristics. The examined motility and oxidative status parameters were as follows:

(i) motility (MOT): percentage of motile spermatozoa (motility > 5 *μ*m/s),
(ii) progressive motility (PROG): percentage of progressive motile spermatozoa (motility > 20 *μ*m/s),
(iii) superoxide dismutase (SOD): a major antioxidant enzyme that catalyses the conversion of two superoxides into oxygen and hydrogen peroxide (H_2_O_2_),
(iv) catalase (CAT): an antioxidant enzyme which degrades H_2_O_2_ to water and oxygen, thereby completing the reaction started by SOD,
(v) glutathione (GLH): the principal nonenzymatic antioxidant specialized in the prevention of oxidative damage to cellular components,
(vi) albumin (ALB): an important nonenzymatic protein with antioxidant properties,
(vii) malondialdehyde (MDA): a byproduct of lipid peroxidation (LPO) and a biomarker indicating the overall oxidative degradation of lipids.

## 2. Materials and Methods

### 2.1. Biological Material

Semen samples were collected in duplicates from 30 Simmental-Fleckvieh breeding bulls kept in the Breeding Centre of the Slovak Biological Services, Nitra, Slovakia, during late spring and early summer 2012. The animals were 4–6 years old and fed a standard diet based on green and cereal fodder, berseem, straw, and concentrated mixtures. Water was supplied constantly. The samples were acquired on a regular collection schedule using an artificial vagina and were immediately transferred to the laboratory. Subsequently, basic semen assessment was performed—volume (mL), pH, and concentration (×10^6^/mL) were evaluated in each sample.

### 2.2. Spermatozoa Motility Analysis

Spermatozoa motility and progressive motility examinations were carried out using the Computer Assisted Semen Analysis (CASA) system—SpermVision (Minitube, Tiefenbach, Germany) with Olympus BX 51 phase contrast microscope (Olympus, Tokyo, Japan). The samples were placed into the Makler Counting Chamber (depth 10 *μ*m, 37 ± 1°C; Sefi Medical Instruments, Haifa, Israel) and immediately assessed. 1000 cells were evaluated in each sample [12].

### 2.3. Sample Processing

The samples were centrifuged (15 min, 10 090 ×g, 4°C) to obtain the cell sediment and seminal plasma fraction. The fractions were separated, and seminal plasma was transferred into 1.5 mL tubes and kept frozen (−80°C). The sediments were moved into tubes containing 1.5 mL distilled water and subsequently lysed via sonication for 40 sec on ice. After sonication, the lysates were centrifuged (15 min, 11 828 ×g, 4°C) to remove cell debris, and the supernatants involving the sperm cell content were transferred into 1.5 mL tubes and stored at −80°C until further analysis [13].

### 2.4. Assessment of Chemical Elements

Both seminal plasma and cell lysates (at least 1 mL) were mineralized by placing the samples in mineralization tubes, adding 2 mL of a nitric and perchloric acid mixture (HNO_3_-HClO_4_; 4 : 1), and heating at 120°C for 65 minutes in a thermostat-controlled digestion block. The resulting solution was diluted to 10 mL with demineralized water. Na and K concentrations were determined by the voltammetric method (ASV) using an EA9C potentiostat model equipped with working CGMDE electrode, AgCl_2_, and platinum electrodes (MTM, Krakow, Poland). Concentrations of Fe, Cu, Mg, and Zn were measured by flame absorption spectrophotometry (FAS) method with the Cole-Parmer 200A model (Cole-Parmer International, Court Vernon Hills, USA). Concentrations are expressed as mg/dL [14].

### 2.5. Biochemical Studies

The measurements were based on a colorimetric reaction of the target substance and a subsequent UV/VIS spectrophotometric detection at a specific wavelength. SOD, CAT, GSH, and ALB were assessed using the Genesys 10 spectrophotometer (Thermo Fisher Scientific Inc., Waltham, USA). MDA content was analysed with the help of the Multiskan FC microplate photometer (Thermo Fisher Scientific Inc., Waltham, USA).

Before any of the prooxidant or antioxidant markers was examined, protein concentration was assessed using the DiaSys Total Protein (DiaSys, Holzheim, Germany) commercial kit. The measurement is based on the Biuret method. When Cu ions (copper sulphate) react with proteins to form a violet blue color complex in alkaline solution, the intensity of the color is directly proportional to the protein concentration and was measured at 540 nm. 

SOD activity was analyzed with the Randox RANSOD assay (Randox Laboratories, Crumlin, Great Britain). This method employs xanthine and xanthine oxidase (XOD) to generate superoxide radicals, which react with 2-(4-iodophenyl)-3-(4-nitrophenol)-5-phenyltetrazolium chloride (I.N.T.) to form a red formazan dye. SOD activity was measured by the inhibition degree of this reaction at 505 nm. The SOD activity is expressed as U/mg protein.

CAT activity was assessed according to Beers Jr. and Sizer [15] by monitoring the decrease of H_2_O_2_ at 240 nm. The calculation was based on the rate of H_2_O_2_ decomposition, which was proportional to the reduction of the absorbance during 1 min. The values are expressed as U/mg protein.

Reduced GSH was determined by the Ellman method [16]. The sample was treated with Ellman's reagent, which interacts with the thiol groups of GSH, cleaving the disulfide bond to give 2-nitro-5-thiobenzoate (NTB^−^) and creating the NTB^2-^ dianion in water at neutral and alkaline pH. This ion has a yellow color and was quantified at 412 nm. GSH concentration was expressed as mg/mg protein.

ALB concentration was measured using the ALB BioLa Test (PLIVA-Lachema, Brno, Czech Republic) commercial kit and expressed as mg/mg protein. The measurement was based on the reaction between ALB and Bromocresol Green at acid pH forming a complex, which was easy to detect photometrically at 578 nm.

MDA content was detected with the help of the TBARS assay, modified for a 96-well plate and ELISA reader. The MDA-TBA product formed by the reaction of MDA and thiobarbituric acid (TBA) under high temperature (90–100°C) and acidic conditions was measured at 530–540 nm [13]. The MDA concentration was expressed as *μ*M.

### 2.6. Statistical Analysis

All data were subjected to statistical analysis using the GraphPad Prism program (version 3.02 for Windows, GraphPad Software incorporated, San Diego, California, USA, http://www.graphpad.com/). Results are quoted as arithmetic mean ± standard error of mean (SEM). Pearson product-moment correlation coefficient analysis for paired samples was used to assess correlations between all examined parameters. Additionally, the samples were categorized in three quality groups according to their motility rates. Comparative analysis of selected parameters in the seminal fractions as well as in the quality groups was carried out by one-way ANOVA with the Bonferroni multiple comparison test. The level of significance for the comparative as well as correlation analysis was set at ^***^(*P* < 0.001); ^**^(*P* < 0.01); ^*^(*P* < 0.05).

## 3. Results and Discussion

Values of the basic seminal parameters are represented in Table 1. Animal donors and samples showed no signs of disease, infection, or pathology. Furthermore, results obtained from routine seminal evaluation met the standards and criteria established for the Simmental-Fleckvieh bovine breed, which is why we ruled out any impact of the health estate on the data obtained from further chemical and biochemical measurements.

Results of the FAS, ASV, and spectrophotometric analyses are shown in Table 2. The concentrations of the chemical elements recorded in both seminal fractions may be expressed in the following descending order: Na > K > Zn > Mg > Fe > Cu [mg/dL]. While the outcomes of the ASV and FAS examinations of the seminal plasma suggest that the concentrations of all elements were within the physiological limits and comparable to the results of other authors [4, 17–19], their concentrations, except for K, were significantly (*P* < 0.001) lower in the spermatozoa lysates. As we found no data available to compare and discuss our detected statistical differences, we assume that unlike the blood plasma, the seminal plasma does not have a proper filtration system and therefore acts as an accumulator of any organic or inorganic substance. At the same time, lower concentrations of chemical elements in the sperm cells prove that spermatozoa lack a typical cytoplasm as a source of potential binding molecules for the minerals. Nonetheless, we have to be aware of the fact that the content of chemical elements within spermatozoa may vary and is highly dependent on the concentration of the sperm cell within the ejaculate.

The seminal plasma is considered to be the central source of antioxidants protecting the seminal components against oxidative damage [10]. Our records agree showing that the LPO expressed as the amount of MDA was significantly higher in the sperm lysates when compared to the seminal plasma (*P* < 0.001; Table 2), suggesting that the spermatozoa lipids are the primary target for FR, while the seminal plasma lipids may be protected by a complex antioxidant system [20]. At the same time, concentrations of GSH and ALB as important FR trapping molecules were significantly lower in the cell lysates (*P* < 0.001; Table 2), proving that the nonenzymatic antioxidant system of the sperm cell is weak and unfavourable [10]. On the other hand, the activity of both antioxidant enzymes was higher in the cell lysates when compared to the seminal plasma (*P* > 0.05 for SOD and *P* < 0.01 for CAT, resp., Table 2). We assume that even though the overall concentration and variability of antioxidants in spermatozoa are lower, their activities may be considerably higher in order to compensate for the lack of antioxidant diversity. 

Tables 3 and 4 display the results of the correlation analysis between the selected motility parameters, biochemical markers, and minerals quantified in bovine seminal plasma and spermatozoa. MOT and PROG as the basic characteristics of spermatozoa viability were significantly (*P* < 0.05; *P* < 0.01; *P* < 0.001) positively correlated with all the enzymatic as well as nonenzymatic antioxidants of both seminal fractions. Inversely, MDA was significantly (*P* < 0.05; *P* < 0.01; *P* < 0.001) negatively correlated with the motility as well as antioxidant characteristics of the seminal plasma and spermatozoa samples. 

The analysis shows that Na, Cu, Fe, Mg, and Zn exhibited positive correlations of various significance with all the motility and antioxidant parameters, as well as significant negative correlations with MDA (*P* < 0.05; *P* < 0.01) in both seminal fractions. Differently, K displayed negative correlations with the seminal and antioxidant characteristics (*P* < 0.05). At the same time, a positive and significant correlation was recorded between K and MDA (*P* < 0.05) in the seminal plasma as well as spermatozoa (Tables 3 and 4). 

To have a better understanding of the results, the samples were categorized in three groups of excellent (Ex; >90% motile; *n* = 11), good (Go; 80–90% motile; *n* = 12) and moderate (Mo; <80% motile; *n* = 7) quality according to their motility rates (Tables 5 and 6). Mean values for MOT and PROG were significantly different between the groups (*P* < 0.05; *P* < 0.001). The highest SOD, CAT, ALB, and GSH values together with the lowest MDA concentration were recorded in the Ex group of both seminal fractions. Inversely, the lowest antioxidant activity but the highest MDA content was detected in the Mo group (Tables 5 and 6). Significant differences (*P* < 0.05; *P* < 0.01) were observed when comparing the prooxidant and antioxidant parameters between the Ex and the Mo groups.

The highest contents of Fe, Cu, Mg, and Zn were recorded in the Ex group; the Mo group exhibited the lowest concentrations of the elements. The highest K content was detected in the Mo group, while its concentration was significantly lower in the Ex group (*P* < 0.01). No significant changes in the Na concentration were detected in the quality groups; however, its highest concentration was revealed in the Ex group, while its lowest concentration was recorded in the Go group.

Our correlation and comparative examinations on typical antioxidants (SOD, CAT, and GSH) confirm their positive effects on spermatozoa motility and quality as well as antioxidant profile concluded in different studies [4, 13, 21–24] and confirm that decreased concentrations and/or activities of FR scavenging molecules are related to a decreased quality, viability, and fertilization potential of semen accompanied by an increased risk of seminal oxidative stress development. Interestingly, ALB, which is not considered to be a dominant protein of the bovine ejaculate [25], exhibited detectable and important concentrations in both seminal plasma and spermatozoa (Table 2). Moreover, positive relationships between ALB, spermatozoa motility, and antioxidant profile confirms the suggestions of Bourdon and Blache [26] as well as Tvrdá et al. [21] that ALB could be an important seminal antioxidant protein acting through its multiple-binding sites and free radical-trapping properties.

Numerous literature sources indicate that LPO could be a primary cause of a decreased seminal quality in fertility disorders [13, 20, 24, 27]. Results of our study demonstrate that the concentration of MDA, a specific marker of LPO, was significantly higher in the low quality seminal fractions and suggest that the activity of oxygen radicals in these samples was the most detrimental (Tables 5 and 6). In addition, our outcomes agree with Tavilani et al. [27] demonstrating strong negative associations between the specific activities of antioxidant enzymes with the MDA content. These observations suggest that seminal CAT and SOD play a significant role in the protection against LPO in ejaculates. 

Semen quality as well as the ability of spermatozoa to undergo capacitation, acrosome reaction, and oocyte fusion is crucial phenomenon intimately related to the antioxidant and mineral profile of the organism. At the same time, seminal chemical elements are directly or indirectly involved in the protection against OS or its modification, induction of changes in the activity of spermatozoa, or interactions with other substances [1].

Despite the fact that Na and K are reported to be indirectly responsible for the maintenance of seminal osmolarity and activity [6], our examinations revealed that while Na exhibited generally favorable effects on the seminal quality and antioxidant balance, K behaved inversely. Gür and Demirci [17] detected a positive impact of Na on all spermatozoa vitality characteristics assuming that Na is crucial for proper physicochemical properties of semen. Our results agree and furthermore conclude that the seminal Na is indispensable for a suitable antioxidant milieu and activity. On the other hand, negative associations between the K concentration, MOT, and PROG (Tables 3 and 4) agree with Gür and Demirci [17] as well as Sheth and Rao [28], proposing that oxygen uptake, glycolysis, and fructolysis could be inhibited by K and indicating that this element may adversely affect spermatozoa activity. Moreover, negative correlations together with high concentrations of K in the Mo groups (Tables 5 and 6) confirm the suggestions of Ford [9] and Griveau et al. [29] that at low pH the K^+^ ion pairs with the superoxide causing a significant increase in LPO and FR formation, which are inversely correlated with sperm motility and antioxidant status, especially with SOD and GSH (Tables 3 and 4), which are directly responsible for superoxide scavenging [11].

Fe and Cu deficiencies represent the most common nutritional problems existing today and are correlated with impaired development and reproductive performance [30]. At the same time, prolonged intake of high doses of both metals may lead to pathological conditions. According to Massányi et al. [3], increased Fe concentration may bear negatively on spermatozoa viability and morphology. Other studies show that mean Fe concentration in seminal plasma was highly associated with sperm progressive motility, gross motility, and viability [18, 31]. Moreover, Fe is integral for catalase, and it has been reported that the CAT activity may be significantly reduced as a result of iron deficiency [32], which explains significant positive associations between Fe and CAT in both seminal fractions (Tables 3 and 4). Incubation of spermatozoa in the presence of Cu had a negative effect on the motility parameters examined by the CASA system in studies by Roychoudhury et al. [8] and Tvrdá et al. [33]. On the contrary, other authors report positive effects of Cu on the sperm count, motility, and progressive motility as well as on the prevention of LPO via the activity of SOD and CAT [4, 31, 34]. Significant positive associations between the seminal plasma Cu and SOD confirm that Cu is essential for a proper activation of the Cu/Zn SOD isoenzyme increasing the number of normal and healthy sperm cells [30, 34]. Differences in the Cu and Fe amounts in the quality groups (Tables 5 and 6) suggest that physiological concentrations of both trace elements may have a stimulating impact on both motility as well as antioxidant status of bovine semen. 

Positive associations between Mg, motility, and antioxidant markers detected in our study are in accordance with Abdul-Rasheed [35] and Eghbali et al. [36]. As shown by Chandra et al. [37], Mg intake decreased LPO and increased SOD as well as CAT activity in rat testicular tissue. Additionally, it has been reported that semen contains secretory granules and vesicles of prostatic origin and containing Mg-dependent ATPase, which might have a regulatory effect on sperm motility by modulating the concentration of essential cations in the seminal environment [38]. Furthermore, significant correlations between Mg and GSH (Tables 3 and 4) may be explained by the fact that GSH is Mg-dependent, as the glutathione synthetase needs Mg^2+^ cations to get activated [39].

Zn levels in seminal plasma have been positively associated with sperm concentration and motility in some studies [40, 41], while nonsignificant to negative correlations have been found in others [42, 43]. Our analysis revealed a significant positive effect of Zn on both spermatozoa motility parameters, as well as antioxidant markers, most significantly with SOD (Tables 3 and 4). The cytosolic Cu/Zn SOD is the major SOD isoenzyme found in seminal plasma as well as spermatozoa [11], which additionally explains positive correlations between Zn and Cu (Tables 3 and 4). Increased FR in infertile males have been previously associated with a decrease of seminal Zn content, arising their harmful effects on the sperm cells which are subsequently linked to abnormal seminal parameters [44], and increase in the oxidation of lipids, DNA, and proteins, causing the loss of spermatozoa membrane integrity [45]. Negative associations between Zn and MDA (Tables 3 and 4), as well as a significant decline of Zn in the quality groups (Tables 5 and 6), may therefore support the evidence defending the antioxidant capacity of seminal Zn.

## 4. Conclusions 

In conclusion, our study presents a complex intercellular and intracellular network of interactions and associations between the mineral elements, markers of spermatozoa quality, and antioxidant profile within the bovine semen. At the same time our results indicate that any mineral imbalance in seminal plasma or spermatozoa may have a negative impact on seminal abnormalities and/or oxidative stress development and therefore may be considered as a risk factor for male fertility complications. At last, we suggest that routine evaluation of chemical composition and antioxidant status of both seminal fractions should become established and standardized for fertility assessment in veterinary practice. 

## Figures and Tables

**Table 1:** Basic seminal and motility characteristics of the samples (*n* = 30).

Parameter	Mean ± SEM
Volume [mL]	7.20 ± 0.50
pH	6.67 ± 0.30
Concentration [×10^6^ cells/mL]	2 995 ± 40.50
Motility [%]	87.37 ± 2.51
Progressive motility [%]	81.79 ± 2.64

SEM: standard error of mean.

**Table 2:** Chemical and biochemical parameters of bovine seminal plasma and spermatozoa examined by the ASV and FAS methods (chemical elements) as well as UV/VIS spectrophotometry (prooxidant and antioxidant markers) (*n* = 30).

Parameter	Seminal plasma	Cell lysates
SOD [U/mg protein]	0.49 ± 0.03	0.60 ± 0.05
CAT [U/mg protein]	1.19 ± 0.10	2.01 ± 0.14∗∗
GSH [mg/mg protein]	0.10 ± 0.02	0.01 ± 0.002∗∗∗
ALB [mg/mg protein]	0.18 ± 0.03	0.10 ± 0.005∗∗
MDA [µM]	2.94 ± 0.19	13.29 ± 0.67∗∗∗
Na [mg/dL]	179.44 ± 7.01	141.11 ± 3.02∗∗
K [mg/dL]	25.97 ± 2.10	21.57 ± 1.15
Fe [mg/dL]	4.15 ± 0.54	2.02 ± 0.30∗∗
Cu [mg/dL]	2.39 ± 0.27	1.15 ± 0.15∗∗
Mg [mg/dL]	7.65 ± 0.90	2.03 ± 0.19∗∗
Zn [mg/dL]	23.59 ± 2.05	9.40 ± 1.10∗∗

Mean ± SEM. ∗∗*P* < 0.01; ∗∗∗*P* < 0.001. SOD: superoxide dismutase; CAT: catalase; GSH: glutathione; ALB: albumin; MDA: malondialdehyde; Na: sodium; K: potassium; Fe: iron; Cu: copper; Mg: magnesium; Zn: zinc.

**Table 3:** Correlations between motility parameters, prooxidant/antioxidant markers, and chemical elements in bovine seminal plasma evaluated by the Pearson product-moment correlation (*n* = 30).

	MOT	PROG	SOD	CAT	GSH	ALB	MDA	Na	K	Cu	Fe	Mg	Zn
MOT	1												
PROG	0.972∗∗∗	1											
SOD	0.583∗∗	0.601∗∗∗	1										
CAT	0.396∗	0.400∗	0.722∗∗∗	1									
GSH	0.440∗	0.452∗∗	0.530∗∗	0.410∗	1								
ALB	0.407∗	0.422∗	0.435∗	0.415∗	0.210	1							
MDA	−0.528∗∗	−0.545∗∗	−0.612∗∗	−0.623∗∗	−0.530∗∗	−0.351∗	1						
Na	0.312∗	0.328∗	0.315	0.304	0.318	0.392∗	−0.365∗	1					
K	−0.387∗	−0.398∗	−0.400∗	−0.209	−0.445∗	−0.365∗	0.346∗	−0.297	1				
Cu	0.423∗	0.441∗∗	0.499∗∗	0.451∗	0.439∗	0.392∗	−0.362∗	0.388∗	−0.305	1			
Fe	0.429∗∗	0.464∗∗	0.470∗∗	0.569∗∗	0.359∗	0.445∗∗	−0.360∗	0.390∗	−0.331∗	0.495∗∗	1		
Mg	0.498∗∗	0.510∗∗	0.475∗∗	0.423∗	0.414∗∗	0.381∗	−0.360∗	0.425∗	−0.215	0.413∗	0.422∗	1	
Zn	0.559∗∗	0.578∗∗	0.532∗∗	0.399∗	0.452∗	0.439∗	−0.482∗∗	0.307	−0.305	0.331∗	0.335∗	0.408∗	1

The interpretation of the results was based on the value of the correlation coefficient: ±0.111–±0.333: low correlation; ±0.334–±0.666: moderate correlation; ±0.667–±0.999: high correlation. ∗*P* < 0.05; ∗∗*P* < 0.01; ∗∗∗*P* < 0.001. MOT: spermatozoa motility [%]; PROG: spermatozoa progressive motility [%]; SOD: superoxide dismutase [U/mg protein]; CAT: catalase [U/mg protein]; GSH: glutathione [mg/mg protein]; ALB: albumin [mg/mg protein]; MDA: malondialdehyde [µM]; Na: sodium [mg/dL]; K: potassium [mg/dL]; Fe: iron [mg/dL]; Cu: copper [mg/dL]; Mg: magnesium [mg/dL]; Zn: zinc [mg/dL].

**Table 4:** Correlations between motility parameters, prooxidant/antioxidant markers, and chemical elements in bovine spermatozoa evaluated by the Pearson product-moment correlation (*n* = 30).

	MOT	PROG	SOD	CAT	GSH	ALB	MDA	Na	K	Cu	Fe	Mg	Zn
MOT	1												
PROG	0.972∗∗∗	1											
SOD	0.620∗∗∗	0.642∗∗∗	1										
CAT	0.425∗	0.440∗	0.789∗∗∗	1									
GSH	0.482∗∗	0.498∗∗	0.585∗∗	0.468∗	1								
ALB	0.437∗	0.450∗	0.461∗	0.430∗	0.251	1							
MDA	−0.520∗∗	−0.539∗∗	−0.699∗∗∗	−0.672∗∗∗	−0.589∗∗∗	−0.461∗	1						
Na	0.345∗	0.359∗	0.355∗	0.349∗	0.339∗	0.322	−0.359∗	1					
K	−0.422∗∗	−0.436∗∗	−0.439∗	−0.245	−0.452∗	−0.366∗	0.336∗	−0.310	1				
Cu	0.440∗	0.454∗∗	0.502∗∗	0.450∗	0.449∗	0.409∗	−0.380∗	0.376∗	−0.311	1			
Fe	0.474∗∗	0.493∗∗	0.469∗∗	0.563∗∗	0.369∗	0.492∗∗	−0.371∗	0.400∗	−0.375∗∗	0.417∗	1		
Mg	0.505∗∗	0.532∗∗	0.512∗∗	0.419∗	0.425∗∗	0.415∗	−0.392∗	0.452∗∗	−0.236	0.433∗	0.369∗	1	
Zn	0.565∗∗	0.590∗∗	0.542∗∗	0.454∗	0.474∗	0.439∗	−0.495∗∗	0.329∗	−0.367∗	0.379∗	0.333∗	0.415∗	1

The interpretation of the results was based on the value of the correlation coefficient: ±0.111–±0.333: low correlation; ±0.334–±0.666: moderate correlation; ±0.667–±0.999: high correlation. ∗*P* < 0.05; ∗∗*P* < 0.01; ∗∗∗*P* < 0.001. MOT: spermatozoa motility [%]; PROG: spermatozoa progressive motility [%]; SOD: superoxide dismutase [U/mg protein]; CAT: catalase [U/mg protein]; GSH: glutathione [mg/mg protein]; ALB: albumin [mg/mg protein]; MDA: malondialdehyde [µM]; Na: sodium [mg/dL]; K: potassium [mg/dL]; Fe: iron [mg/dL]; Cu: copper [mg/dL]; Mg: magnesium [mg/dL]; Zn: zinc [mg/dL].

**Table 5:** Average values of seminal motility parameters, prooxidant/antioxidant markers, and chemical elements in bovine seminal plasma quality groups (Mean ± SEM) and Bonferroni multiple comparison test results.

	Ex (*n* = 11)	Go (*n* = 12)	Mo (*n* = 7)
MOT [%]	93.75 ± 4.50	85.63 ± 3.88^∗a^	63.20 ± 3.99^∗∗∗b;∗∗∗c^
PROG [%]	90.67 ± 4.57	81.75 ± 1.33^∗a^	59.33 ± 6.01^∗∗∗b;∗∗∗c^
SOD [U/mg protein]	0.68 ± 0.04	0.51 ± 0.05	0.31 ± 0.05^∗∗b;∗c^
CAT [U/mg of protein]	1.59 ± 0.04	1.28 ± 0.03	1.10 ± 0.02
GSH [mg/mg of protein]	0.12 ± 0.06	0.08 ± 0.007	0.06 ± 0.006^∗b^
ALB [mg/mg of protein]	0.23 ± 0.04	0.18 ± 0.05	0.17 ± 0.03
MDA [*μ*M]	1.54 ± 0.37	2.18 ± 0.15	2.96 ± 0.46^∗b^
Na [mg/dL]	189.60 ± 5.76	164.40 ± 4.33	183.60 ± 5.87
K [mg/dL]	23.10 ± 4.90	29.40 ± 5.13	31.50 ± 4.45^∗b^
Fe [mg/dL]	4.09 ± 0.86	3.74 ± 0.61	3.39 ± 0.84
Cu [mg/dL]	2.37 ± 0.08	2.12 ± 0.08	2.05 ± 0.22
Mg [mg/dL]	8.62 ± 1.03	7.29 ± 0.98	5.72 ± 0.97^∗∗b^
Zn [mg/dL]	29.07 ± 2.08	21.76 ± 2.12^∗a^	15.63 ± 2.10^∗∗b^

^a^Ex versus Go; ^b^Ex versus Mo; ^c^Go versus Mo. ∗*P* < 0.05; ∗∗*P* < 0.01; ∗∗∗*P* < 0.001. Quality groups are based on the motility values: excellent quality (>90% motile), good quality (80–89% motile), and moderate quality (<79% motile). MOT: motility; PROG: progressive motility; SOD: superoxide dismutase; CAT: catalase; ALB: albumin; GSH: glutathione; MDA: malondialdehyde; Na: Sodium; K: potassium; Fe: iron; Cu: copper, Mg: magnesium; Zn: zinc.

**Table 6:** Average values of seminal motility parameters, prooxidant/antioxidant markers, and chemical elements in bovine spermatozoa quality groups (Mean ± SEM) and Bonferroni multiple comparison test results.

	Ex (*n* = 11)	Go (*n* = 12)	Mo (*n* = 7)
MOT [%]	93.75 ± 4.50	85.63 ± 3.88^∗a^	63.20 ± 3.99^∗∗∗b;∗∗∗c^
PROG [%]	90.67 ± 4.57	81.75 ± 1.33^∗a^	59.33 ± 6.01^∗∗∗b;∗∗∗c^
SOD [U/mg protein]	0.78 ± 0.07	0.65 ± 0.06	0.40 ± 0.07^∗∗b;∗c^
CAT [U/mg of protein]	2.37 ± 0.24	2.05 ± 0.23	1.65 ± 0.25^∗b^
GSH [mg/mg of protein]	0.02 ± 0.006	0.01 ± 0.007	0.005 ± 0.001^∗b^
ALB [mg/mg of protein]	0.13 ± 0.04	0.09 ± 0.005	0.06 ± 0.005^∗b^
MDA [*μ*M]	8.74 ± 1.17	14.10 ± 1.15^∗a^	16.46 ± 1.36^∗∗b;∗c^
Na [mg/dL]	129.60 ± 4.76	114.35 ± 4.33	126.93 ± 4.87
K [mg/dL]	18.10 ± 2.90	23.95 ± 3.13	30.79 ± 3.45^∗∗b;∗c^
Fe [mg/dL]	2.39 ± 0.86	2.09 ± 0.61	1.51 ± 0.21^∗b^
Cu [mg/dL]	1.37 ± 0.12	1.02 ± 0.10	0.80 ± 0.09^∗b^
Mg [mg/dL]	2.65 ± 0.50	1.99 ± 0.40	1.49 ± 0.27^∗b^
Zn [mg/dL]	15.07 ± 2.08	10.76 ± 1.13^∗a^	7.63 ± 0.926^∗∗b^

^a^Ex versus Go; ^b^Ex versus Mo; ^c^Go versus Mo. ∗*P* < 0.05; ∗∗*P* < 0.01; ∗∗∗*P* < 0.001. Quality groups are based on the motility values: excellent quality (>90% motile), good quality (80–89% motile), and moderate quality (<79% motile). MOT: motility; PROG: progressive motility; SOD: superoxide dismutase; CAT: catalase; ALB: albumin; GSH: glutathione; MDA: malondialdehyde; Na: Sodium; K: potassium; Fe: iron; Cu: copper, Mg: magnesium; Zn: zinc.
